# MicroRNA in Glioblastoma: An Overview

**DOI:** 10.1155/2017/7639084

**Published:** 2017-11-06

**Authors:** Barbara Banelli, Alessandra Forlani, Giorgio Allemanni, Anna Morabito, Maria Pia Pistillo, Massimo Romani

**Affiliations:** ^1^Laboratory of Tumor Epigenetics, Ospedale Policlinico San Martino, Genova, Italy; ^2^Department of Health Sciences, University of Genova, Genova, Italy

## Abstract

Glioblastoma is the most aggressive brain tumor and, even with the current multimodal therapy, is an invariably lethal cancer with a life expectancy that depends on the tumor subtype but, even in the most favorable cases, rarely exceeds 2 years. Epigenetic factors play an important role in gliomagenesis, are strong predictors of outcome, and are important determinants for the resistance to radio- and chemotherapy. The latest addition to the epigenetic machinery is the noncoding RNA (ncRNA), that is, RNA molecules that are not translated into a protein and that exert their function by base pairing with other nucleic acids in a reversible and nonmutational mode. MicroRNAs (miRNA) are a class of ncRNA of about 22 bp that regulate gene expression by binding to complementary sequences in the mRNA and silence its translation into proteins. MicroRNAs reversibly regulate transcription through nonmutational mechanisms; accordingly, they can be considered as epigenetic effectors. In this review, we will discuss the role of miRNA in glioma focusing on their role in drug resistance and on their potential applications in the therapy of this tumor.

## 1. Introduction

Epidemiological studies estimate that more than 250,000 new cases of central nervous system (CNS) tumors worldwide are expected every year with variable incidence rates ([1] and http://www.cancer.gov/types/brain/hp/adult-brain-treatment-pdq). Although glioblastoma is considered a rare tumor (Orphanet 360), it accounts for 4% of all cancer deaths making it as one of the deadliest human tumors. According to the current classification [2], approximately 38% of these tumors are at high grade (WHO III, anaplastic astrocytoma or AA and WHO IV, glioblastoma or GB) and hence are virtually lethal.

Given the extremely limited success of the standard treatment in prolonging survival in GB patients, considerable efforts were undertaken to develop targeted therapies that could significantly improve the outcome of these patients [3]. In this respect, epigenetics and epigenetic modulators have become a preferred field of investigation because of their influence in many aspects of cancer [4, 5].

Epigenetics, at large, is the mechanism utilized by living cells to decode and utilize properly the information contained in the raw DNA sequence. In practice, epigenetics consists in a “code” that lays on top of the genetic code and translates the simple information into function [6]. By definition, epigenetics does not change the “content of the information” (i.e., the sequence) and acts through reversible modifications like cytosine methylation at CpG doublets, postsynthetic modifications of the histones (acetylation, methylation, phosphorylation, etc.), and changes in the chromatin conformation. In the last years, a new class of effectors has been added to the epigenetic machinery: the microRNAs or in general, the noncoding RNAs that are capable of reversibly interfering with the transcription and translation of the genes without altering DNA sequence as expected for an epigenetic mechanism.

In this review, we will discuss some general aspects of miRNA in glioma focusing on the circuitry between miRNA and other epigenetic determinants like DNA methylation in this tumor, their role in drug resistance, and their potential therapeutic implications.

## 2. Epigenetics and Epigenetic Networks in Glioblastoma

Transcriptional profiling has delineated four major molecular subtypes of glioblastoma that could be better characterized by mutational, copy number variation, and methylation analyses [7–9]. In particular, this classification defines two clinical groups of GBs with distinct treatment response and outcome (Table 1). Overall, GB could be classified as “primary,” a group that includes three transcriptional subtypes (classical, mesenchymal, and neural) and “secondary” glioblastoma derived from the evolution of low-grade GB that include the transcriptional proneural subtype and that can be loosely subdivided in two subgroups according to the mutational and chromosomal status. The average survival is 31 months for secondary glioblastoma and only 15 months for the patients with primary GB. As can be seen in Table 1, the major features that distinguish primary from secondary GB can be considered, at large of epigenetic nature, namely, *MGMT* methylation status and the CpG island methylator phenotype (CIMP).

One of the first evidences of the primary role of epigenetic mechanisms in GB was the discovery of the effect of the inactivation by methylation of the *MGMT* gene on the sensitivity to the alkylating agent temozolomide (TMZ). In two seminal papers published in 2005 [10, 11], Stupp et al. and Hegi et al. established that the inactivation of the *MGMT* gene by DNA methylation in the tumor is associated with better survival in GB patients treated with TMZ and radiotherapy compared to the patients with unmethylated *MGMT*. Hypermethylation of *MGMT* occurs also in primary GB; however, it is a characteristic feature of secondary glioblastoma and is the “iceberg tip” of a more extensive alteration of the DNA methylation status known as “CpG island methylator phenotype” or CIMP.

The concept of CIMP was developed in 1999 by Toyota et al. that showed the concomitant presence of hypermethylation at many different CpG sites in a subset of colorectal cancer patients with distinct clinicopathological characteristics and favorable outcome [12]. Since then, CIMP was described in many other tumors (for a recent review, see [13]) although it is not clear if the CIMP phenotype is tissue-specific or if all CIMP+ tumors represent a class of tumors with similar characteristics. Moreover, the molecular parameters, including the methylation cut-off levels and the genes to be considered to positively assess the presence of CIMP in a given tumor are not well established. The clinical characteristics of CIMP+ tumors differ; indeed, it differs in GB [8], colon carcinoma [14], myeloid leukemia [15], and breast cancer [16]. The CIMP+ phenotype is a predictor of better outcome whereas in other tumors like neuroblastoma [17, 18] and melanoma [19], CIMP+ tumors are associated with poor prognosis. A possible explanation for the nonuniversal clinical significance of CIMP is the absence of accurate criteria that define CIMP so that the true phenotype of the tumor cannot be always assessed.

In GB, the CIMP phenotype clearly distinguishes the primary (CIMP−) from the secondary (CIMP+) tumors and is tightly associated with inactivating mutations of the *IDH1* and *IDH2* genes [8]. The mechanistic link between *IDH* mutations and CIMP was discovered in 2012 when it was demonstrated that IDH1 is an epigenetic controller that modulates the pattern of histone and DNA methylation. This occurs through the inhibition by D-2-hydroxyglutarate of the jumonji histone lysine demethylases (KDM) and of the TET-hydroxylases that convert 5-methylcytosine into 5-hydroxyl-methylcytosine thus leading to the accumulation of 5-methylcytosine.

Recently, histone modifications also have gained importance in GB and the possibility of pharmacological intervention on histone deacetylases (HDAC) has been exploited [20]. Moreover, the involvement of *KDM* genes in GB development and drug resistance has been demonstrated [21–23].

MicroRNAs, a class of noncoding RNAs, are considered epigenetic modifiers because they control the functionality of the genome by base pairing of nucleotides 2–8 of their sequence to the 3'UTR of mRNA forming the so-called “silencing complex” [24]. Since their inhibitory function is sequence-specific and does not involve the permanent alteration of the DNA sequence, miRNAs are considered an integral part of the epigenetic machinery.

In glioblastoma, as in many other tumors, the remodeling of the epigenome is an important aspect of the biology of the tumor [25, 26] and the interaction between epigenetic factors and the cell signaling cascade appears as a promising target for new therapeutic approaches [20, 23, 25, 27, 28].

## 3. The Interplay between Tumor Suppressing and Oncogenic miRNA in Glioblastoma

From the current release, 21 of the miRNA database lists 2588 mature and 1881 precursor human miRNA sequences (http://www.mirbase.org/cgi-bin/browse.pl?org=hsa). Each of these miRNA can modulate the expression of several mRNAs, and each mRNA can be modulated by several miRNA generating an extraordinary complex regulatory network. In a literature survey of miRNA deregulated in GB, it was found that the majority of them (*n* = 253) were overexpressed compared to normal brain tissue, 95 were downmodulated, and conflicting results were reported for 17 of them [29].

The genes targeted by deregulated microRNAs in GB are involved in many pathways including cell proliferation, resistance to apoptosis, autophagy, invasion and metastasis, angiogenesis, and drug resistance. Since microRNAs have multiple targets in different tissues, they may have oncogenic (oncomiR) or antioncogenic effects depending upon the biological context.

Several miRNAs acting as tumor suppressor genes have been identified; some of them are unique of glioblastoma whereas others are involved also in other tumors. In principle, all miRNA interfering with the histone methyltransferase *EZH2* (Table 2) can be considered as tumor suppressors, in particular let-7 which inhibits also oncogenes like *MYC* and *K*-*RAS* [30, 31] and is capable of inhibiting glioblastoma cell proliferation [32]. miR-128 and miR-34a are two examples of miRNA acting as tumor suppressor in glioblastoma. miR-128 is an antiproliferative miRNA that interferes with multiple pathways targeting genes involved in glioblastoma pathogenesis like *EGFR* and *PDGFRA* [33] and *WEE1* [34] and *E2F3a* [35]. miR-34a interferes with cell proliferation through multiple targets (*CDK6*, *CCND1*, *NOTCH*, and others). When the functionality of miR-34a is restored, this miRNA acts as a tumor suppressor gene reducing cell proliferation and invasion [36]. MiR-124 and miR-137 are two microRNA significantly downregulated in high-grade gliomas and *in vitro* can induce phenotypic changes, growth arrest, and differentiation in glioma stem cells and thus can be considered oncosuppressive miRNA [37]. Most deregulated miRNAs in GB interfere with cell proliferation pathways, particularly those of *EGFR* and *AKT*. A prototype of this group of miRNA is miR-7 whose transfection in GBM cells leads to decreased invasiveness and increased apoptosis fulfilling the basic requirements of a tumor suppressor [38, 39].

Many miRNAs are upregulated in glioblastoma and can be functionally classified as oncomiR. Historically, miR-21 was the first oncogenic miRNA to be identified [40] and can target a set of oncosuppressor genes including *PTEN* [41] and the metalloproteinase inhibitor *TIMP3* which is involved in extracellular matrix remodeling, tumor infiltration, and angiogenesis [42, 43]. Thus, miR-21 targets an entire network of tumor suppressor genes and its inhibition by complementary oligonucleotides blocks GB cell growth *in vitro* and *in vivo* [44]. It is reasonable to hypothesize that the delivery of an inhibitor of miR-21 at the tumor site might be a useful addition to the standard therapy.

The targeting of multiple oncosuppressor genes easily explains the oncogenic mechanism of miR-21. On the contrary, the oncogenic properties of miR-221and miR-222, overexpressed in a variety of tumors including GB, have several oncogenic functions including the inactivation of the cell cycle suppressors p27 and p57 [45, 46].

Apoptosis and autophagy are two mechanisms utilized to eliminate dysfunctional or otherwise stressed cells, and resistance to apoptosis is one of the hallmarks of cancer cells. Unsurprisingly miRNA can target several genes at the center of both mechanisms. Oncogenic antiapoptotic miRNAs like mir-21 [42], miR-221/mir-222 [47], and miR-335 [48] are overexpressed in glioblastoma and interfere with the p53/Bcl-2/PUMA and TGF-*β* signaling (miR-21/miR-221/miR-222) or with the potential tumor suppressor *DAAM1* (miR-335). Conversely, proapoptotic oncosuppressive miRNAs like miR-218 and miR-451 are downmodulated in GB [49, 50]. Interestingly, downmodulation of miR-221/miR-222 restores the p53 pathway, activates apoptosis, and sensitizes GB cells to TMZ [51]. In addition to its antiapoptotic effect, miR-21, along with miR-17, inhibits autophagy. Downregulation of these miRNA increases the sensitivity of GB cells to TMZ and radiation expanding the cell population undergoing apoptosis [52, 53].

Experimental models indicate that in GB exists a subpopulation of cells possessing the characteristics of neural stem cells that are responsible for continuous proliferation and drug resistance [54, 55]. miRNA profiling revealed that glioma cells have an expression profile remarkably similar to that of embryonic and neural precursor cells and distinct from that of a normal adult brain [56]. Interestingly, a set of 71 miRNA deregulated in human spontaneous GB is remarkably similar to that of chemically induced mouse glioma suggesting a common pattern of cancer development [56]. The miRNAs deregulated in GB and in neural precursor cells are clustered in seven genomic regions and have been associated with many other cancers like the mir-17 family cluster [57–59], miR-182-183 cluster [60], miR-302-367 and miR-372 [59, 61, 62], and the Dlk1 domain [63].

In GB, recurrent chromosomal aberrations are lacking; nevertheless, chromosomal instability (CIN) is considered an important mechanism for the establishment and maintenance of tumor heterogeneity [64]. CIN has the dual function of responding to various stressing conditions while being, at the same time, the origin of further genome destabilization. The comparison by genome-wide analysis between long-survival GB outlier patients (OS > 33 months) and short-term survivors (OS < 7 months) showed significantly lower genetic alterations in the short-term survivors than in long-term survivors [65]. The increased genomic instability of long-surviving patients might be responsible for the increased vulnerability of the cells to the standard radio- and chemotherapy. Along with this line, it was shown that glioma stem cells have high CIN that accounts for maintaining tumor heterogeneity and that increasing CIN further abolishes tumorigenicity as if an upper limit of genetic instability exists to initiate tumor formation [66].

Unsurprisingly, miRNAs are implicated in the molecular mechanisms of CIN and the intervention of these molecules into chromosomal instability has been studied in several tumors but, to the best of our knowledge, not yet in glioblastoma. Several miRNAs, like miR-26a and miR-28-5p, interfere with genes involved in cell replication and cell cycle checkpoints [67, 68] while others like miR-1255b, miR-148b^∗^, and miR-193b^∗^ reduce homologous recombination at G1 thus maintaining genomic stability [69]. Other miRNAs like miR-214 [70], miR-137 [71], miR-1255b, miR-148b^∗^, and miR-193b^∗^ [69] regulate at a different level the DNA repair mechanisms.

## 4. miRNA Targeting Immune Checkpoints and Inflammatory Molecules in Glioblastoma

**Figure 1:**
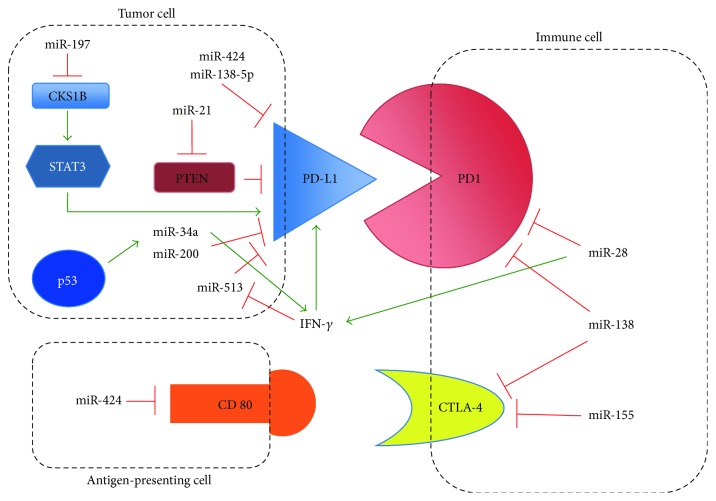
Interactions between miRNA and immune checkpoints. This nonexaustive scheme shows the major interactions between miRNA and immune checkpoint molecules. Red and green arrows indicate the suppressive or activating interactions, respectively.

Targeting the immune checkpoint gene PD1, its ligand PDL1, and other genes like CTLA-4 has raised considerable attention in the recent years because of the dramatic antitumor effect exerted by antibodies against these molecules particularly in tumors with limited therapeutic options like lung cancer and melanoma (reviewed in [72, 73]). In this respect, glioblastoma is not an exception and GB escapes T-cell killing by activating immune checkpoints [74]. In an experimental model of glioma, the blockade of three immune checkpoints (IDO, CTLA-4, and PD1) significantly increased the survival of tumor-bearing mice [75]. However, these findings might not be generally applicable because of the extreme heterogeneity of this tumor and the absence of solid predictive biomarkers of sensitivity to immune checkpoints inhibitors [76]. Immune checkpoints can be modulated by microRNAs [77], and Figure 1 summarizes some of the interaction of the complex network between miRNA, genes, and immune checkpoints.

Intuitively, this complex network requires an extremely precise tuning since immune checkpoint molecules can be blocked by a given miRNA (e.g., miR-34a and miR-138) that, at the same time, can indirectly promote the expression of cytokines that in turn induce the expression of the same checkpoint molecules that are targeted by the miRNA.

Microglia and astrocytes, along with macrophages, are part of the glioma microenvironment, astrocytes are part of the brain immune system as they express cytokines and chemokines, and glioma cells produce and are targets of inflammatory molecules [78, 79]. Glioma cells produce IL-1 which is a potent inducer of angiogenesis and invasion and in glial cells, strongly upregulates miR-155 implicated in inflammation-mediated cancer development [80]. Besides miR-155, other IL-1-induced miRNAs involved in inflammation, miR-21, and miR-146 are upregulated in gliomas [81]. Interestingly, miR-146 is a negative regulator of astrocyte-mediated inflammation [82], and upregulation of this miRNA decreases the expression of its target TRAF6 that is linked to seizure frequency in glioma patients suggesting that miR-146 could be involved in the epileptogenic focus surrounding the tumor [81].

## 5. Invasiveness and the Blood Brain Barrier as Escape Mechanisms from Therapy

An important mechanism contributing to the failure of treatment in GB is the invasiveness of the tumor. Brain is a particular environment that is made impermeable to external molecules by the blood brain barrier (BBB); this prevents the efficient targeting of glioma cells with antineoplastic drugs unless the BBB is severely damaged as in glioma above 2–4 mm [83]. Nevertheless, GB can escape treatment also because of its capacity to infiltrate the brain forming niches in regions where the BBB is intact. Invasiveness is part of the epithelial to mesenchymal transition (EMT), a mechanism through which cells lose the epithelial phenotype and acquire that of mesenchymal cells. Several miRNAs are involved in EMT or in general into the mechanisms of invasion; they include miR-21, miR-146, miR-10b, and miR-7 that target directly or indirectly metalloproteinase inhibitors [84, 85], adhesion molecules, and other genes involved in metastasis and cell invasion [86–88].

GB is a highly vascularized tumor, and this feature likely contributes to the invasive and proliferative capacity of the tumor and to the shielding of GB cells behind the BBB. A set of miRNA (miR-296, miR-125b, and others) can directly or indirectly fine-tune angiogenic factors and promote neoangiogenesis in GB [89, 90]. In GB, angiogenesis can be stimulated also by other mechanisms like hypoxia through the targeting of *HIF3A* by miR-210-3p that results in the overexpression of VEGF [91]. Interference with VEGF is not the only angiogenic mechanisms exerted by miRNA in glioma; indeed, neovascularization can be promoted by miR-93 that targets integrin-*β*8 involved in cell-cell and cell-matrix interactions [92]. The transport mechanisms of miRNA across the BBB is still debated and could involve extracellular vesicles (EV) like exosomes that could serve as a communication tool in nonpathologic situations [93] or between tumor cells and their environment and trigger cell proliferation [94].

MicroRNAs have also an important role in drug and radioresistance as will be described in another part of this review.

## 6. Circulating miRNA as Glioma Biomarkers

Circulating nucleic acids and circulating tumor cells [95, 96] are extensively exploited as tumor markers to predict outcome and to monitor the response to therapy. Importantly, in GB, response to therapy often results in enhancement of the captation of the contrast medium that can be disguised as progression (“pseudoprogression”) challenging the imaging assessment of the disease [97]. The distinction between true progression and pseudoprogression is a diagnostic need required for optimizing patient's care.

Overall, both blood and cerebrospinal fluid are a source of circulating biomarkers and relevant glioma mutations like those of *IDH1/IDH2* can be detected in circulating DNA [98]. On the contrary, circulating tumor cells are so scarce in glioma that, with the available technologies, their clinical potentials remain an open issue. Similarly, serum proteomics has not yet provided clinically useful results [99].

Extracellular vesicles (EV) are an attractive source of circulating biomarkers because they act as a cargo for many types of molecules that are protected from degradation [100]. EV are released by all cells to enable the communication between nonadjacent cells, and EV secretion is an early response of cancer cells to a variety of stress conditions including treatment [101]. Interestingly, EV are not randomly loaded and their content seems to reflect the biology of the donor cells making EV an ideal source of circulating biomarkers [102]. Although the utilization of EV in the clinical context is still in its infancy, promising results were obtained in two small GB trials. One of these was aimed at comparing the level of the DNA repair enzymes *MGMT* and *APNG* in the parental tissue and in EV before and after TMZ treatment [103]. In the second study, exosome mRNA was examined to study the changes of expression of immune markers and cytokines after inoculation of a tumor vaccine in glioblastoma patients [104]. Both studies demonstrate that in principle, molecules carried by EV can be utilized to develop robust assays to monitor disease progression in glioblastoma.

Different studies reported the miRNA profiling in the plasma of glioma patients or evaluated the level of defined circulating miRNA known to be involved in this tumor. A restricted signature of eleven miRNAs was selected through a systematic review of the literature and utilized to screen a small cohort of GB patients, and the results showed that the level of expression of miR-497 and miR-125b could distinguish between low- and high-grade glioma [105]. In principle, these types of markers could be very useful to monitor the evolution of primary low-grade glioma into secondary GB for better timing the beginning of therapeutic procedures. The expression of miRNA deregulated in GB was included in several studies on circulating biomarkers. Indeed, miR-21 was found overexpressed in plasma of glioblastoma patients compared to normal controls while miR-128 and miR-342-3p were downmodulated in the same set of patients [106]. Expression of these miRNA returned to baseline levels after treatment suggesting that circulating miRNA can be utilized to monitor disease response to treatment and disease relapse [106]. Interestingly, a recent whole miRNA profiling from the plasma of a relatively large set of glioblastoma patients identified a signature predicting disease-free and overall survival independently from other clinicopathological factors [107].

In conclusion, there are strong indications that circulating biomarkers have the potential to recapitulate the molecular complexity of GB and that they could gain clinical relevance. Nevertheless, more informative biomarkers are needed to develop robust and reproducible assay before a liquid biopsy could become a standard clinical practice.

## 7. MicroRNA and DNA Methylation: Interplay between Epigenetic Factors

The effects of miRNA dysregulation have been extensively studied initially at the level of single interaction between miRNA and its target gene or genes in a countless number of pathologic and physiologic conditions and more recently as components of signatures or within functional pathways. Intriguingly, miRNA can also be subjected to epigenetic control through DNA methylation and histone modifications [108] thus establishing a complex interplay capable of interfering, directly or indirectly, with multiple pathways in extraordinary complex networks that have been partially explored in several tumors including glioblastoma [109–115].

The effect of miRNA on epigenetic modifier genes and the influence of DNA methylation on miRNA expression in glioblastoma have been studied in some detail. In particular, targeting of *DNMT3a* and *DNMT3b* by miRNA-29, miRNA-29a, and miRNA-148 was observed, and it is generally believed that this interaction may contribute to the general hypomethylation seen in cancer [116–118]. However, the direct link between the expression of the miRNA-29 family and of miRNA-148 and the methylation status of glioblastoma cells has not yet been studied.

EZH2 is a histone methyltransferase that catalyzes the trimethylation of H3 at lysine 27 (H3K27), a postsynthetic modification of H3 leading to transcriptional inactivation [119]. Furthermore, EZH2 promotes the de novo DNA methylation interacting with DNMT3A and DNMT3B [120]. In glioblastoma, and other tumors, EZH2 is overexpressed and acts as an oncogene with multiple mode of actions including cell invasion utilizing largely tumor-specific mechanisms [121, 122], cell cycle progression, maintenance of cell stemness [123], and, last but not least, the development of drug resistance [123, 124] and inhibition of apoptosis [119]. It thus appears that EZH2 is at the center of many cancer-related pathways and that it must be kept under stringent transcriptional control. Several miRNAs, reported in Table 2, and lncRNAs are integral components of mechanisms that regulate *EZH2* expression; however, the role of some of them in GB has not yet been investigated or experimentally proven.

Although manipulating EZH2 expression may seem a promising and logical strategy for the therapy of GB and other tumors, it must be reminded that knocking down a gene that masters DNA and histone methylation will epigenetically influence a vast number of genes with unpredictable effects. Indeed, it was shown that prolonged inhibition of EZH2 results in GB tumor progression whereas short-term inhibition improves survival in animal models [125]. However, it is likely that the major benefits from EZH2 inhibition will derive from appropriate scheduling of cytotoxic and epigenetic drugs as recently proposed [27].

Acetylation and deacetylation of histones H3 and H4 are postsynthetic modifications that contribute to the switching between permissive (acetylated) and repressed (deacetylated) conformation of the chromatin [126]. Acetylation and deacetylation are driven by two sets of enzymes: histone acetyltransferase (HAT) and histone deacetylases (HDAC) that include several variants. In glioblastoma, the expression of *HDAC1* and *HDAC3* is inversely correlated with survival of GB patients, whereas that of *HDAC4, HDAC5*, *HDAC6*, and *HDAC11* is positively correlated with survival of glioma patients [127]. *HDAC1* is a known target of miR-449 and miR-874 [128, 129] but the clinical relevance of the expression pattern of these miRNA in GB is not known. *HDAC4* is targeted by miR-1 and miR-155 [130, 131]; in contrast with the *HDAC* expression data, exogenously expressed miR-1 that putatively should interfere with *HDAC4* acts as a tumor suppressor gene prolonging survival in an animal model [132]. On the contrary, the expression of miR-155 in glioma is prevalent in high-grade tumors with a worse prognosis [133].

Several other genes belonging to the epigenetic machinery are targeted by miRNA; their involvement in glioblastoma is not yet well established, and they will not be discussed here.

Besides controlling epigenetic modifier genes, miRNA can be subjected to epigenetic control. This control can be exerted at three levels: DNA methylation, histone modification, and combined DNA methylation and histone modification. Approximately half of the known miRNAs are hosted in CpG -rich regions and are thus potential targets of DNA methylation; indeed, the effect of DNMT inhibitors has been tested on several cancer cells showing the activation of the miRNA-target gene axis [114, 115, 134]. To the best of our knowledge, the systematic analysis of miRNA silenced by DNA methylation in glioma has not yet been performed; nevertheless, several examples of miRNA silenced by DNA methylation in GB have been described along with the functional effects of their reexpression [135].

miR-211 targets *MMP9*, activates the caspase-9/caspase-3 apoptotic cascade, and was found to be hypermethylated in GB [136]. miR-204, methylated and downregulated in glioma, when activated suppresses the expression of stem transcription factor SOX4, reduces cell invasion, and prolongs survival in animal models [137]. miR-23 is hypermethylated in GB and is reactivated by 5-Azacytidine treatment leading to cell cycle arrest [138]. miR-145 is underexpressed in astrocytoma compared to normal brain, functionally acts as a tumor suppressor gene targeting *SOX2*, a stem-maintaining gene, and reduces proliferation and migration of GB cells targeting CTGF and NEDD9 [135, 139, 140]. miR-137 is epigenetically inactivated in many cancers, and its expression is diminished in GB and in glioma stem cells. Reexpression of miR-137, hypermethylated in GB tumor samples, promotes neural differentiation and decreases the expression of stem cell markers (Oct4, SOX2, and Nanog) [141]. Furthermore, miR-137 is also an inhibitor of EZH2 [142]. In one of the most comprehensive methylation analysis of miRNA promoter regions to date [143], 29 miRNAs differentially methylated in high-grade glioma were identified. The hypermethylation (and low expression) of three of them, miR-155, miR-210, and miR-355, was a strong predictor of better outcome and longer PFS. However, upon validation in different patient series and in multivariate models, only miR-155 remained of prognostic value independently from other indicators like histology, *MGMT* methylation, and *IDH1*/*IDH2* mutation. Therefore, miR-155 can be considered both as an oncomiR in GBM with multiple biological roles including the activation of the NFkB pathway [143]. On the other hand, miR-155 could act also on the immune cell compartment by downmodulating the immune checkpoint molecule CTLA-4 exerting the function of a tumor suppressor miRNA (Figure 1 and [144]). miR-181c, another example of miRNA downregulated by epigenetic mechanisms in glioblastoma, targets the NOTCH2 pathway and is important in self-renewal, proliferation, and invasion of GB cells [145]. This miRNA was sorted out by chromatin immunoprecipitation/sequencing screening as a region containing H3K4me3 and H3K27ac marks partially overlapping with a CpG-rich region close to miR-181c that is hypermethylated in GB [146].

## 8. miRNA and Chemo- and Radioresistance in Glioblastoma

The response to treatment in GB patients is variable and probably depends from tumor heterogeneity that originates from genetic and epigenetic alterations which can influence the behavior of the disease. In this respect, the relation between miRNA and chemo- and radiotherapy has been extensively exploited to search for new possible therapeutic targets or to predict and improve the response to treatment.

Earlier preclinical studies showed that cisplatin could increase the efficacy of TMZ by decreasing the activity of MGMT [147] but several clinical trials have tested the activity of cisplatin in GB patients with limited success [148, 149]. Indeed, GB cells after an initial and positive response to cisplatin develop chemoresistance. Many biological pathways underlie the resistance to cisplatin and platinum derivatives [150], and several miRNAs contribute to the reduction of platinum sensitivity. Let-7b seems to be involved in cisplatin resistance affecting the cyclin D1 pathway [151], and miR-873, which targets Bcl-2, is downregulated in a time-dependent manner by cisplatin and, if overexpressed, increases apoptosis in cisplatin-resistant GB cells [152].

Temozolomide is, at the moment, the first-line drug for high-grade glioma treatment independently from the methylation status of *MGMT* (https://www.cancer.gov/types/brain/hp/adult-brain-treatment-pdq#link/_1089_toc). Several mechanisms of resistance to TMZ have been identified (reviewed in [153]), and epigenetic mechanisms, besides *MGMT* methylation, are explored as possible effectors of constitutive or acquired TMZ resistance in GB patients. In this respect, a substantial body of evidence gained mostly in preclinical models supports the idea that many miRNAs interfere with the response of the cells to TMZ.

As discussed above, miR-21 is consistently upregulated in astrocytic tumors (grade II–IV) [154] and downmodulates an entire set of oncosuppressor genes [41, 155, 156]. Indeed, miR-21 has antiapoptotic activity in glioblastoma cells [40] and treatment of GB cells with TMZ results in miR-21 overexpression while its inhibition with specific anti-miR-21 results in high apoptotic levels upon treatment with TMZ [157].

The *AEG-1* (astrocyte elevated gene-1), overexpressed in GB tumor samples, favors the infiltration capabilities of established GB cell lines [158], and its downmodulation by siRNA sensitizes the cells to TMZ. *AEG-1* is directly targeted by miR-136 that, when exogenously overexpressed, increases the cytotoxic activity of TMZ [159]. In principle, the expression of miR-136 could be utilized as an indicator of drug response in GB patients.

Direct targeting of genes controlling the apoptotic pathway is another mechanism capable to modulate TMZ resistance in GB cells. For example, miR-139 inhibits the expression of the antiapoptotic gene *Mcl-1*, a member of the *Bcl-2* family, and sensitizes GB cells to the effect of TMZ [160]. Similarly, miR-143 targets several genes involved in the pathogenesis of cancer like *K-* and *N-RAS*, *Bcl*-*2*, and *IGF*-*IR*. The overexpression of miR-143 sensitizes GB cells to apoptosis induced by TMZ and inhibits invasion and proliferation, and this effect has been attributed to the direct targeting of *N-RAS* and, indirectly, to the dephosphorylation of *AKT* and to the downmodulation of *HIF* and *VEGF* as a result of N-*RAS* inhibition [161].

A more direct link with TMZ resistance is attributed to miRNA targeting directly *MGMT*. The inhibition of *MGMT* through different mechanisms besides DNA methylation silencing may at least partly explain the positive response to treatment in patients without methylation of *MGMT*. In this respect, miR-603 and miR-181d directly target and independently coregulate *MGMT* inducing sensitivity to TMZ [162].

As mentioned in a previous section of this review, miR-29c is a direct inhibitor of the de novo DNA methyltransferases *DNMT3a/DNMT3b* and is an indirect suppressor of *MGMT* via silencing of Sp1, a *MGMT* transcription factor. Interestingly, forced expression of miR-29c, which is downmodulated in glioblastoma, sensitizes cells to TMZ [163].

Along with chemotherapy, radiotherapy is an integral part of the clinical management of GB and different miRNAs are involved in radiosensitization or radioresistance.

Low levels of ATM protein are a major determinant of radiosensitivity in glioblastoma, and *ATM* is the target of different miRNAs such as miR-100 and miR-26a. High level of miR-100 expression was found in the radiosensitive glioma cell line; on the other hand, its ectopic expression in radioresistant cells downmodulates ATM and sensitizes the cells to ionizing radiation [164]. Ionizing radiation induces ATM expression (and radioresistance), and miR-26a restores radiosensitivity by targeting ATM [164]. It thus appears that drug and radioresistance in GB are controlled by an array of miRNA that directly or indirectly interferes with multiple pathways involved in drug and radiation response.

## 9. miRNA and Innovative Therapies in Glioblastoma

The development of multiomics strategies has led to impressive advancements of the knowledge on the mechanisms behind cell transformation and has opened the possibility of selectively targeting cancer cells in many types of tumors including GB [165–168].

In principle, a drug-based “biologic therapy” is aimed at changing the cell phenotype through the use of molecules capable of blocking well-defined pathways. This can be achieved either through the functional inhibition of the enzymatic activities of a given protein or through the ablation of the protein itself. The first strategy leaves the protein unmodified while the second acts on the expression of the target protein and, in theory, should be more effective.

Transcriptional inhibition of a given gene can be obtained by RNA interference, a mechanism originally described in worms [169] and later in higher organisms [170–172]. In practice, it was observed that double-stranded RNA delivered into the cells caused the degradation of the target mRNA and this system is now widely employed for the transient or stable gene inactivation. MicroRNA, because of their hairpin and partially complementary structure, can be considered as an endogenous form of interfering RNA that depending on the extent of complementarity with their targets can either stop the translation or promote the degradation of the mRNA.

A major question to be answered is if miRNA modulation of gene transcription is powerful enough to have a therapeutic consequence in glioma also in view of the necessity of obtaining an adequate delivery at the tumor site. While *in vitro* assays demonstrated the feasibility of this approach, the *in vivo* translation of these studies appears a much more complex task. The partial knowledge of the miRNA networks, pathways, target genes, and of their interplay in healthy and diseased cells adds further difficulties to the short-term therapeutic utilization of these strategies.

One of the questions that need to be answered is if miRNA has the potential to enter the routine clinical practice. Along with this review, we have seen that suppressing certain oncomiR (i.e., miR-21) or inducing the expression of tumor suppressor miRNA like let-7 has dramatic effects on cell behavior and suppress GBM viability. Nevertheless, many major issues still remain, first of all, the problem of delivery, and also the choice between monospecific synthetic siRNA and polyspecific miRNA mimics or miRNA antagonists. siRNAs have the obvious advantage to selectively target specific pathway components while miRNA can interfere with multiple pathways at once. However, the off-target effects of the miRNA have to be carefully evaluated. Furthermore, if a siRNA cocktail seems a reasonable tool, the utilization of a miRNA cocktail seems more complex also because of the conspicuous off-target effect of this cocktail and because of the interactions between different miRNAs [114].

### 9.1. Biological Therapies in GB: The Delivery Issue

In Glioblastoma, the presence of the BBB represents a major challenge to the utilization of miRNA in therapy because if the BBB is damaged and permeable at the tumor site, its integrity is maintained at the infiltrating tumor areas that are those responsible for tumor relapse after initial surgery and radiochemotherapy [83]. Nevertheless, some preliminary results support the use of antago-miR or miRNA mimics in the therapy of glioma although the issue of the active concentration that can be achieved at the tumor site needs to be taken into consideration.

The ideal goals of the delivery across the BBB are as follows:

- to increase the local drug concentration
- to increase the possibility of using drugs that do not pass through the BBB
- to increase the possibility of reaching the tumor niches surrounded by integral BBB that are responsible for tumor relapse
- to increase the possibility of using antitumor drugs in low-grade glioma protected by a functional BBB

Delivery systems can be passive or active. The objective of the passive methods is the permeabilization of the BBB with hyperosmotic agents, surfactant, ultrasounds, and electromagnetic waves to transiently open the tight cell-cell junctions of the BBB [173]. In this respect, a randomized phase III clinical trial showed that the combined treatment of TMZ and pulsed electric fields is superior to the standard TMZ treatment [174]. The direct infusion of drugs or other bioactive molecules at the site of the lesion after craniotomy, even if highly selective, was found of limited utility because of the poor diffusion in the perilesional area where the tumor niches are [175].

Active transport toward the lesion is considered, in general, a more efficient mode to selectively deliver drugs or other molecules within the brain. The most promising active delivery systems are those based on nanoparticles of less than 200 nm [176] that carry on their surface molecules that can be recognized by specific receptors on the BBB, like transferrin, lactoferrin, transferrin receptor, and glutathione [177].

The most commonly utilized carriers for drug delivery in the CNS are liposomes at a single or double layer of approximately 100 nm of diameter that are engineered with molecules for tumor targeting [178, 179]. Some liposomal formulations have entered into the clinical practice, and others are being tested in clinical trials [179–183].

Other utilized delivery systems are the polymeric colloids (PDP) [184–186] or other colloidal formulations (LNC) [187–189] that can be modified to pass the BBB and to target the tumor utilizing two ligands [185]. The delivery systems based on nanoparticles are highly promising but their toxicity, biocompatibility, and payload retention must be carefully evaluated [190, 191].

### 9.2. Targeting Glioblastoma Cells with miRNA

Convention-enhanced delivery, a drug delivery method based on catheters stereotactically implanted to infuse the treatments directly to the tumor site, was utilized to deliver let-7a into the brain of mice xenografted with an aggressive GB. This treatment was well tolerated and was effective in reducing the expression of *HMGA2*, one of the targets of let-7a [192].

Although direct delivery of miRNA into the brain seems to be effective, intuitively non- or minimally invasive drug delivery methods may be preferable. In this respect, nanoparticles seem a very promising strategy and were exploited to deliver at the tumor site not only a variety of drugs but also miRNA [193]. In principle, nanoparticles should overcome the poor systemic stability of oligonucleotides and improve their delivery; as said above, different nanoparticle formulations are available each with advantages and disadvantages but they can all be engineered to target the tumor site and, in the case of brain tumors, to transit the BBB. To date, the most common carriers are targeted liposomes of 100 nm [178, 194] that are being tested in animal models [180, 181].

Several types of nanoparticles have been utilized to carry a number of miRNA and to test their biological effects. For example, antago-miR-21 carried by RNP were utilized to successfully rescue the expression of antioncogenic *PTEN* and of *PDCD4* and to promote tumor regression in a model system [195]. Similarly, antago-miR-21, delivered by poly(lactic-*co*-glycolic acid) (PLGA), sensitizes the effect of TMZ *in vitro* [196].

Another interesting example of cooperative treatment in glioblastoma is provided by a multifunctional delivery system MSNPs (mesoporous silica nanoparticle) charged with TMZ molecules and decorated by an anti-miR-221 PNA-octaarginine conjugate (R8-PNA221) that increases the biological effect of TMZ in drug-resistant cells [197]. A similar effect was seen with miR-34a encapsulated in a polyglycerol scaffold [198].

Mesenchymal stem cells (MSC) are an interesting and potentially very effective method to target sites of injury or of inflammation and tumors for therapeutic purposes [199, 200], and it was demonstrated that functional miRNA can be conveyed to neural progenitor cells by cocultivation with appropriately engineered MSC [201].

As mentioned above, miR-10b is involved in tumor invasion and is an optimal therapeutic target because of its high and generalized expression in all GB subtypes [86–88]. A preclinical in vivo study focused on the inhibition of miR-10b in an orthotopic GB xenograft model compared the results of different delivery methods utilizing as endpoint the inhibition of the tumor growth [202]. Brain injections, systemic injections, and intracranial osmotic pumps were compared, and each one showed weak and strong points. The antagonist of miR-10b administered by the three routes resulted in the inhibition of miR-10b and in turn reactivated its target genes, attenuated tumor growth, and prolonged survival. Considering the possible translation from the bench to the bedside, the systemic injections of miR-10b inhibitor were less invasive compared to the other routes and had minimal or no side effects on extracranial tissues and with a good delivery through the BBB.

miRNA “sponges” are oligonucleotide sequences that contain many binding sites for a specific miRNA or miRNA family and act as competitive inhibitors of the binding of the miRNA to their targets [203]. The utility of these “sponges” in GB was recently demonstrated for miR-23b in an orthotopic *in vivo* model and showed the reduction of angiogenesis, migration and invasion, and in turn the malignancy of the tumor [204]. Circular RNAs (circRNA) are natural examples of sponges that are highly resistant to degradation and that are now subject of in-depth investigations because of their strong regulatory activity on miRNA [205].

## 10. Conclusions

MicroRNAs are epigenetic regulatory molecules that possessing multiple targets have a profound impact on cell physiology and pathology. MicroRNAs are players of the “epigenetic orchestra” that fine-tune the coordinate transcription of the genetic information. It is quite clear that control exerted by miRNA is extraordinary complex, that indeed a single miRNA can bind many genes, and that each gene can be recognized by many miRNAs in an extremely complex direct and indirect regulatory circuitry. Obviously, mastering this network could have dramatic effects on cell behavior.

Therefore, it is not surprising that although our knowledge of the complex effects and interactions between miRNA and genome is still incomplete, the potential implications of miRNA for the diagnosis and prognosis, for the patients' stratification and for their personalized therapy, were not overlooked. However, in order to translate the impressive basic knowledge so far gained on miRNA onto the clinical practice, several issues urgently need to be addressed. Besides the technicisms of delivery and targeting, the major problem remains that of understanding the miRNA effects not anymore at the level of single miRNA-target interaction, but utilizing a “holistic” approach to fully appreciate the balance between miRNA and target genes of opposite functions.

It is quite clear that this will be a highly demanding, but exciting, task for the scientists of the immediate future.

## Figures and Tables

**Table 1:** Molecular characteristics of glioblastoma subtypes according to methylation, expression, mutation, copy number variations patterns, and clinical outcome.

*IDH1/IDH2* status	Wild type			Mutated	
*MGMT*	Mostly unmethylated			Methylated	
Methylation status	CIMP−			CIMP+	
Mutations	*TERT*			*ATRX-TP53*	*CIC-FUBP1*
CNV	EGFR+	NF1−		PDGFRA+	PDGFRA+; 1p19q−
Molecular subtype	Classical	Mesenchymal	Neural	Proneural	
Outcome	Very poor outcome			Significantly improved outcome	Significantly improved outcome. Better response to TMZ than 1p19q+

**Table 2:** miRNA involved in the regulation of EZH2.

miRNA	Action	Reference
Let-7a	Direct targeting of EZH2 in nasopharyngeal carcinoma, inhibition of glioma growth by targeting K-RAS	[206, 207]
miR-26a	Inhibits growth of nasopharyngeal carcinoma targeting EZH2	[208]
miR-101	miR-101 downregulation in GB results in EZH2-induced proliferation regulating the methylation status of CPBE1	[209, 210]
miR-124	Modulates the proliferation of epatocarcinoma cells by direct targeting of EZH2	[211]
miR-138	Blocks GB tumorigenicity by EZH2-CDK4/6-pRb-E2F1 signaling cascade	[212]
miR-214	Targeting of EZH2 in skeletal muscles	[213]
miR-708	Inhibits GB cell proliferation targeting EZH2, AKT1, MMP2, CCND1, Parp-1, and Bcl-2	[214]
